# Stability of the gut microbiota in persons with paediatric-onset multiple sclerosis and related demyelinating diseases

**DOI:** 10.1177/13524585221079533

**Published:** 2022-03-16

**Authors:** Geoffrey Liang, Feng Zhu, Ali I Mirza, Amit Bar-Or, Charles N Bernstein, Christine Bonner, Jessica D Forbes, Morag Graham, Janace Hart, Natalie C Knox, Ruth Ann Marrie, Julia O’Mahony, Gary Van Domselaar, E Ann Yeh, Yinshan Zhao, Brenda Banwell, Emmanuelle Waubant, Helen Tremlett, the Canadian Paediatric Demyelinating Disease Network

**Affiliations:** Division of Neurology, Faculty of Medicine, The University of British Columbia, Vancouver, BC, Canada/The Djavad Mowafaghian Centre for Brain Health, Vancouver, BC, Canada; Division of Neurology, Faculty of Medicine, The University of British Columbia, Vancouver, BC, Canada/The Djavad Mowafaghian Centre for Brain Health, Vancouver, BC, Canada; Division of Neurology, Faculty of Medicine, The University of British Columbia, Vancouver, BC, Canada/The Djavad Mowafaghian Centre for Brain Health, Vancouver, BC, Canada; Center for Neuroinflammation and Experimental Therapeutics and Department of Neurology, Perelman School of Medicine, University of Pennsylvania, Philadelphia, PA, USA; Department of Internal Medicine, Max Rady College of Medicine, Rady Faculty of Health Sciences and University of Manitoba Inflammatory Bowel Disease Clinical and Research Centre, University of Manitoba, Winnipeg, MB, Canada; National Microbiology Laboratory, National Microbiology Laboratory, Public Health Agency of Canada, Winnipeg, MB, Canada; Roy Romanow Provincial Laboratory, Regina, SK, Canada/Department of Pathology and Laboratory Medicine, University of Saskatchewan, Saskatoon, SK, Canada; National Microbiology Laboratory, National Microbiology Laboratory, Public Health Agency of Canada, Winnipeg, MB, Canada/Department of Medical Microbiology and Infectious Diseases, Max Rady College of Medicine, Rady Faculty of Health Sciences, Winnipeg, MB, Canada; Department of Neurology, University of California San Francisco, San Francisco, CA, USA; National Microbiology Laboratory, National Microbiology Laboratory, Public Health Agency of Canada, Winnipeg, MB, Canada/Department of Medical Microbiology and Infectious Diseases, Max Rady College of Medicine, Rady Faculty of Health Sciences, Winnipeg, MB, Canada; Department of Internal Medicine, Max Rady College of Medicine, Rady Faculty of Health Sciences, Winnipeg, Manitoba, Canada; The Hospital for Sick Children, University of Toronto, Toronto, ON, Canada; National Microbiology Laboratory, National Microbiology Laboratory, Public Health Agency of Canada, Winnipeg, MB, Canada/Department of Medical Microbiology and Infectious Diseases, Max Rady College of Medicine, Rady Faculty of Health Sciences, Winnipeg, MB, Canada; The Hospital for Sick Children, University of Toronto, Toronto, ON, Canada; Division of Neurology, Faculty of Medicine, The University of British Columbia, Vancouver, BC, Canada/The Djavad Mowafaghian Centre for Brain Health, Vancouver, BC, Canada; Division of Neurology, Children’s Hospital of Philadelphia, Perelman School of Medicine, University of Pennsylvania, Philadelphia, PA, USA; Department of Neurology, University of California San Francisco, San Francisco, CA, USA; Division of Neurology, Faculty of Medicine, The University of British Columbia, Vancouver, BC, Canada/The Djavad Mowafaghian Centre for Brain Health, Vancouver, BC, Canada

**Keywords:** Multiple sclerosis, demyelinating disease, gut microbiota, stability, paediatric, monophasic acquired demyelinating syndrome

## Abstract

**Objective:**

Examine if the gut microbiota composition changes across repeated samples in paediatric-onset multiple sclerosis (MS) or monophasic-acquired demyelinating syndromes (monoADS).

**Methods:**

A total of 36 individuals (18 MS/18 monoADS) with ⩾2 stool samples were included. Stool sample-derived DNA was sequenced. Alpha/beta diversities and genus-level taxa were analysed.

**Results:**

Mean ages at first sample procurement (MS/monoADS) = 18.0/13.8 years. Median time (months) between first/second samples = 11.2 and second/third = 10.3. Alpha/beta diversities did not differ between stool samples (*p* > 0.09), while one genus – *Solobacterium* did (*p* = 0.001).

**Conclusions:**

The gut microbiota composition in paediatric-onset MS and monoADS exhibited stability, suggesting that single stool sample procurement is a reasonable first approach.

## Introduction

Many gut microbiota-related studies in multiple sclerosis (MS) have typically relied on procuring a single sample from participants.^1^ Little is known about whether this is sufficient.

Individuals with paediatric-onset MS and related diseases represent unique opportunities to explore the gut microbiota early on in the disease process, such as the recently demonstrated relationship between the gut microbiome (using metagenomics) and disease activity.^2^ We assessed if the gut microbiota composition remained stable across repeated stool samples (⩾2) procured over the short-term in individuals with paediatric-onset MS and monophasic-acquired demyelinating-syndromes (monoADS).

## Method

Individuals with symptom onset <18 years and no antibiotic use ⩽30 days prior, diagnosed with MS (McDonald criteria, 2017) or monoADS who provided ⩾2 stool samples were eligible. See Supplementary Data (Methods) for details. Included individuals were enrolled from six sites across Canada and one in the United States (November/2015-March/2018).

Cohort characteristics were captured by trained coordinators using standardized forms and questionnaires were completed at home by parents/participants.

Stool samples were shipped on ice, stored at −80^o^C, then DNA was extracted, amplified and sequenced (V4 hypervariable region, 16S rRNA gene, via the Illumina MiSeq platform), and clustered into amplicon sequence variants (ASVs) using Deblur via QIIME2 (Quantitative Insights Into Microbial Ecology; v.2019.1).^3^ The gut microbiota’s composition was assessed using alpha (Shannon) and beta (unweighted UniFrac) diversity metrics.

Characteristics of the MS and monoADS participants were described. Alpha diversity was compared between the first and second stool samples using the pairwise Wilcoxon signed-rank test and between all three samples using the Friedman test. Unweighted UniFrac was displayed using the first two components from principal coordinates (PC) analysis and confidence ellipsoids. Permutational multivariate analysis of variance (PERMANOVA) was used to compare between stool samples. Phylum-level relative abundances were summarized. The most important genus-level taxa were identified using feature volatility analysis.^4^ The top five genera were analysed for variability between stool samples using linear mixed effects models with sample number (first, second, third) as the response variable, including a participant-specific random intercept, adjusted for age at stool sample, sex and diagnosis (MS/monoADS).^4^ Longitudinal similarity of all samples was compared between MS/monoADS participants using the non-parametric microbial interdependence test (NMIT).^4^ Complementary analyses (MS cases only) explored whether any change in disease-modifying therapy (DMT) use associated with microbiome changes (alpha diversity). QIIME2 v2020.11, R v4.0.1 and SPSS v23.0 were used for analyses.^3,5,6^ Informed consent/assent and ethics were obtained (Supplementary file).

## Results

**Figure 1.**
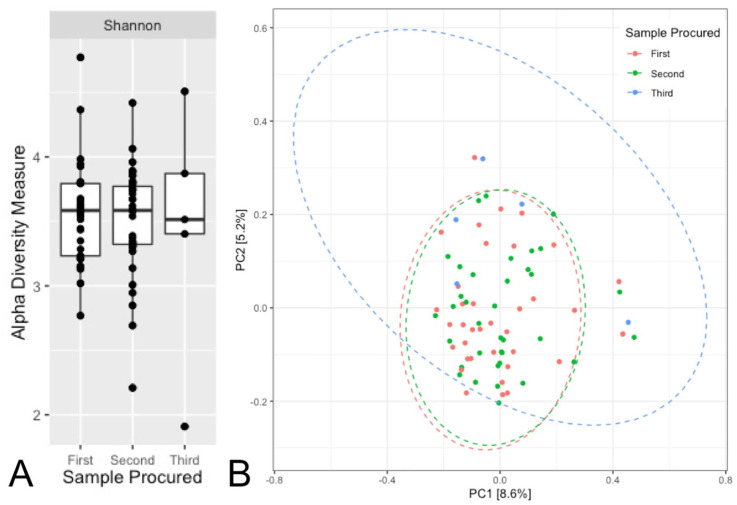
Alpha (Shannon) and beta (principal coordinates analysis of unweighted UniFrac) diversities of the gut microbiome over time for the entire cohort (multiple sclerosis and monophasic acquired demyelinating syndrome participants combined) for all stool samples procured. Key: for Panels A and B, *n* = 36 participants contributed at least two stool samples, and 5 contributed three samples. The mean time between the first and second samples was 11.0 months, and between the second and third was 8.2 months. Panel A: the Wilcoxon signed-rank test was performed when comparing the first and second stool samples procured and the Friedman test when comparing across all three stool samples. Wilcoxon signed-rank *p* = 0.441, Friedman *p* = 1.000. Panel B: the principal coordinates (PC) 1 and 2 are the two axes that represent the most variability and are shown in percentages. Ellipsoids show the 95% confidence intervals for the corresponding sample number. PERMANOVA *p* = 0.983.

A total of 36 individuals provided 77 stool samples (all participants provided 2, 5 of them provided 3) over a 26-month period. The median time (months) between the first/second sample = 11.2 (range = 2.0–25.0) and second/third = 10.3 (range = 1.8–13.6). Participant characteristics, including the Bristol stool scale, body mass index (BMI) and diet, did not differ significantly between timepoints (Table 1, Wilcoxon signed-rank/Friedman *p* > 0.05; second/third sample-related characteristics not shown). Alpha and beta-diversity did not differ significantly between samples for the entire cohort or MS cases alone (Wilcoxon signed-rank/Friedman *p* > 0.09; PERMANOVA *p* > 0.9, Figures 1, Supplementary Figure E1–E2). The most abundant phyla were the *Firmicutes* and *Bacteroidetes*, followed by *Actinobacteria, Proteobacteria, Verrucomicrobia* and *Lentisphaerae* (Supplementary Figure E3). From the feature volatility analyses and linear mixed models, the only genus that changed (increased) significantly over time was *Solobacterium* (adjusted *p* = 0.001). Genus-level NMIT results indicated that longitudinally, the MS and monoADS participants’ gut microbial composition did not differ statistically from each other (*p* = 0.289, Supplementary Figure E4). Alpha diversity did not differ significantly among the four MS participants who stopped/started/switched DMT between their first/second stool samples (Wilcoxon signed-rank *p* > 0.14, Supplementary Figure E5).

**Table 1.** Cohort characteristics for the paediatric-onset multiple sclerosis (MS) and monophasic acquired demyelinating syndromes (monoADS) participants, primarily at the first stool sample (with select characteristics shown between the first and second stool samples).

Characteristic at first stool sample, *n* (%) unless stated otherwise		MS cohort, *n* = 18		monoADS cohort, *n* = 18	
Sex, female		13 (72%)		8 (44%)	
Age at disease onset, years: mean (SD; range)		14.4 (3.9; 4.3–17.9)		7.6 (4.0; 0.6–13.9)	
Age at sample collection, years: mean (SD; range)		18.0 (5.0; 5.4–27.4)		13.8 (4.8; 5.8–24.5)	
Disease duration at sample collection, years: mean (SD; range)		3.6 (3.7; 0.2–10.6)		6.0 (2.6; 2.1–11.2)	
Self-identified race	White	13 (72%)		13 (72%)	
	Non-White	5 (28%)		3 (17%)	
	Unknown	0		2 (11%)	
Country of residence	Canada	12 (67%)		17 (94%)	
	United States	6 (33%)		1 (6%)	
Disease modifying therapy^a^	Never (pre-stool sample)	2 (11%)		18 (100%)	
	Glatiramer acetate	7 (39%)		N/A	
	Interferon beta	8 (44%)		N/A	
	Dimethyl fumarate	3 (17%)		N/A	
	Natalizumab	1 (6%)		N/A	
	Rituximab	1 (6%)		N/A	
	Fingolimod	1 (6%)		N/A	
Comorbidities^b^	Asthma	4 (22%)		4 (22%)	
	Atopic dermatitis	2 (11%)		3 (17%)	
	Other^c^	9 (50%)		10 (56%)	
	None	8 (44%)		8 (44%)	
Stool samples included: total number		39		38	
Participants with exactly 2 samples		15		16	
Participants with exactly 3 samples		3		2	
Time between first and second sample, months: median (range)		9.4 (2.0–25.0)		12.2 (8.7–21.6)	
Characteristic at first and second stool sample, *n* (%) unless stated otherwise		First stool sample	Second stool sample	First stool sample	Second stool sample
Body mass index: mean (SD; range)		21.5 (3.8; 13.8–29.8)	22.0 (4.2; 15.7–32.1)	20.4 (4.7; 14.0–30.0)	20.7 (5.1; 14.0–32.1)
Bristol stool scale	Median (range)	4 (2–6)	4 (1–6)	3 (2–6)	3 (1–5)
	Hard (1–2)	3 (17%)	2 (11%)	6 (33%)	4 (22%)
	Medium (3–5)	10 (55%)	14 (78%)	11 (61%)	14 (78%)
	Loose (6–7)	3 (17%)	1 (6%)	1 (6%)	0
	Unknown	2 (11%)	1 (6%)	0	0
Block Kids Screener: dietary intake per day^d^, median (SD; range)	Energy (kcal/day)	980.4 (317.5; 625.3–1767.8)	935.3 (639.8; 566.7–3052.9)	1221.7 (591.8; 566.7–2715.6)	1222.1 (732.6; 566.7–3632.6)
	Carbohydrate (g/day)	132.0 (39.3; 66.9–216.6)	124.8 (70.8; 63.0–320.4)	145.5 (82.1; 63.0–371.6)	117.6 (70.9; 52.9–316.3)
	Fat (g/day)	41.6 (14.2; 22.4–70.5)	39.6 (32.8; 22.0–150.3)	50.4 (20.2; 22.9–95.2)	45.2 (36.5; 24.2–179.2)
	Protein (g/day)	48.3 (16.7; 22.7–70.5)	39.5 (25.6; 17.45–111.0)	51.2 (32.5; 24.1–164.2)	52.0 (38.5; 24.1–193.2)
	Fibre (g/day)	10.5 (3.8; 3.0–16.5)	9.0 (6.7; 3.0–30.3)	10.0 (5.9; 6.3–24.9)	9.0 (6.9; 4.2–32.3)

MS: multiple sclerosis; SD: standard deviation.

Key: All ranges refer to the minimum and maximum values. Any missing data are explicitly shown in the table as ‘unknown’.

a Some participants were exposed to more than one disease modifying therapy before the first stool sample; thus, the total percentages may exceed 100%.

b Some participants had more than one comorbidity; percentages do not add up to 100%.

c Examples of other comorbidities included acne (one MS case), attention deficit hyperactivity disorder (one MS), depression/anxiety (one MS, one monoADS) and febrile seizures (one MS).

d A total of two monoADS participants were not included in the summaries, as total daily caloric intake was reported to be <500 kcal/day.

## Discussion

We examined the longitudinal stability of the gut microbiota in a cohort of individuals with paediatric-onset MS and monoADS across at least two, and up to three stool samples procured over 26 months. Neither alpha nor beta diversities differed significantly between stool samples procured from participants at up to three different points in time, with most samples procured 9–12  months apart. Furthermore, longitudinally, the overall genus-level composition of the gut microbiota samples (procured from the same individual) did not differ between the individuals with MS compared with those with monoADS. We can infer that, at least in the short-term, the gut microbiota composition in paediatric-onset MS and monoADS participants was relatively stable.

We also examined the five most important genera, as selected by feature analysis, and found that the only genus that changed (increased) significantly over time was *Solobacterium*. This genus belongs to the *Clostridium* cluster XVI, which has been associated with MS.^1,7^ *Solobacterium moorei*, the only species identified in the *Solobacterium* genus, in concert with other microbes, has been implicated as a potential driver of a proinflammatory gut microenvironment.^7^

We were unable to find another published study that examined whether the gut microbiota is stable or fluctuates over time in a paediatric-onset MS or monoADS population. One study did report (primarily descriptively) stability in the gut microbiota in 102 adult participants with/without various immune-mediated diseases (19 had MS), at least across 2 samples procured over 2 months.^8^

Others have also reported relative stability of the gut microbiota over time in healthy adults. The first study procured up to 4 stool samples from 9 participants over 3 months; authors reported minimal within-person variability in gut microbiota composition.^9^ A second study spanned 68 months, with 2–13 stool samples procured from 37 participants; authors reported overall stability with >70% strain similarity between samples.^10^

To the best of our knowledge, our study is the first to explore the stability of the gut microbiota in paediatric MS and related demyelinating diseases. Paediatric-onset MS remains relatively rare such that our sample size was modest, and we had access to a limited number of samples per participant. It would be of value for future studies to assess larger cohorts of individuals, with more samples collected at different time intervals and over longer periods of time. Trends observed in our study could potentially become significant in larger studies.

Nonetheless, our study findings are reassuring and help inform the design and analyses of future gut microbiota studies in MS and monoADS. Particularly when resources are finite and participant burden is prohibitive, our findings provide preliminary evidence to suggest that analysis of single samples from participants is a reasonable approach.

## Supplemental Material

sj-docx-1-msj-10.1177_13524585221079533 – Supplemental material for Stability of the gut microbiota in persons with paediatric-onset multiple sclerosis and related demyelinating diseases Click here for additional data file. Supplemental material, sj-docx-1-msj-10.1177_13524585221079533 for Stability of the gut microbiota in persons with paediatric-onset multiple sclerosis and related demyelinating diseases by Geoffrey Liang, Feng Zhu, Ali I Mirza, Amit Bar-Or, Charles N Bernstein, Christine Bonner, Jessica D Forbes, Morag Graham, Janace Hart, Natalie C Knox, Ruth Ann Marrie, Julia O’Mahony, Gary Van Domselaar, E Ann Yeh, Yinshan Zhao, Brenda Banwell, Emmanuelle Waubant and Helen Tremlett in Multiple Sclerosis Journal
