# Supplementary material for: Stability of the gut microbiota in persons with paediatric-onset multiple sclerosis and related demyelinating diseases
Source: Mult Scler. 2022 Mar 16;28(11):1819–24. doi: 10.1177/13524585221079533 (PMC9442770; doi:10.1177/13524585221079533)
Supplement: sj-docx-1-msj-10.1177_13524585221079533 – Supplemental material for Stability of the gut microbiota in persons with paediatric-onset multiple sclerosis and related demyelinating diseases [file sj-docx-1-msj-10.1177_13524585221079533.docx]

Supplementary Data

**Additional methods**

Study population:

The cohort were all participants enrolled in the Canadian Pediatric Demyelinating Disease Network (CPDDN) study ^1,2^. Participants were invited to provide a stool sample at each study visit, as part of the wider CPDDN study.^1,2^ Visits were typically scheduled annually, with up to three visits occurring during the study period. MonoADS was defined as an initial acute clinical episode of symptoms involving the CNS, with evidence of inflammatory demyelination and with no new clinical or MRI findings of recurrent demyelination^3^ (confirmed after a median observation period of 62 months from first symptom onset to first stool sample, range from 26 to 134 months in our included cohort of 18 monoADS participants).

Cohort characteristics:

The Block Kids Food Screener was used for data collection on nutritional intake and the Bristol Stool Scale was used to measure stool type.^4,5^ We summarized nutrition intake per day as total energy (calories) and grams for carbohydrates, fats, proteins, and fibre. The Bristol Stool Scale was categorized into hard (types 1-2), medium (3-5), and loose (6-7). Medication use (including supplements) were coded according to the World Health Organization’s (WHO) Anatomical Therapeutic Chemical (ATC) classification system, level 4 (chemical subgroup). Participants reporting implausible daily caloric intake (<500 or >5000 calories per day) were excluded in any diet-related estimates.^6^

Bioinformatics:

ASVs were assigned taxonomy using a Naïve Bayes classifier trained on SILVA (v.132) via QIIME2 and examined at the phylum and genus-level. Additional alpha and beta diversities were analysed for the entire cohort combined, and then MS cases only, using the same methods as outlined in the main manuscript.

Statistical analyses:

Bristol stool scale, BMI, and nutritional data were analysed using the Wilcoxon signed-rank and Friedman tests. For the complementary analyses (MS cases only), any change in DMT use was considered, including any change in starting, stopping or switching any DMT between stool samples.

Ethics and Data availability:

Informed consent or assent was obtained from individual participants and their parent/legal guardian. The relevant research ethics boards at each institution approved the study (the Hospital for Sick Children, the Universities of: British Columbia, Calgary, Manitoba, McGill, Memorial, and Western). Authors can be contacted for data access; requests will be assessed on a case-by-case basis, based on the scientific rigor of the proposed research question.


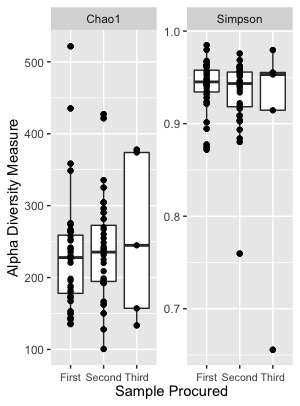


**Figure E1**. Additional alpha diversity metrics for the gut microbiome over time for the entire cohort (multiple sclerosis and monophasic acquired demyelinating syndrome participants combined) for all stool samples procured.

**Key**:

A total of n=36 participants contributed at least two stool samples, and 5 contributed three samples. The mean time between the first and second samples was 11.0 months, and between the second and third was 8.2 months. The Wilcoxon signed-rank test was performed when comparing the first and second stool samples procured and the Friedman test when comparing across all three stool samples.

Chao1 (Wilcoxon signed-rank *P* = 0.226, Friedman *P* = 0.449); Simpson (Wilcoxon signed-rank *P* = 0.093, Friedman *P* = 0.819).


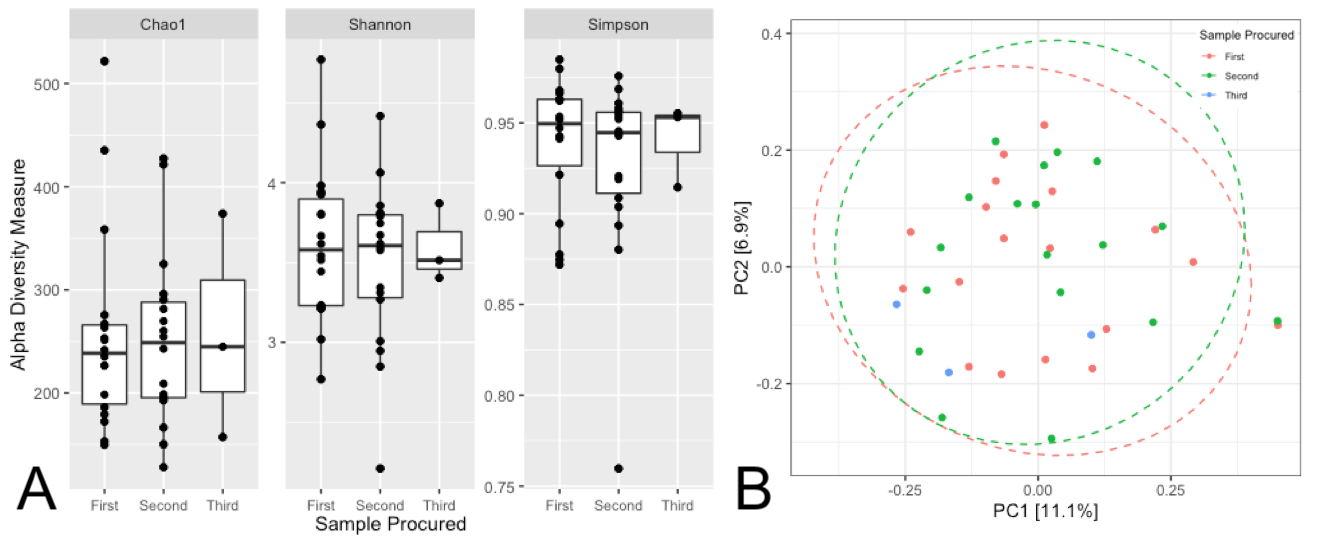


**Figure E2**. Alpha and beta diversity-related analyses for the multiple sclerosis (MS) participants only, for all stool samples procured.

**Key**: All n=18 MS participants contributed at least two stool samples, and 3 contributed three samples. The mean time between the first and second sample was 9.5 months, and between the second and third was 6.3 months.

The Wilcoxon signed-rank test was performed when comparing the first and second stool samples procured and the Friedman text when comparing all three stool samples.

Panel A. Alpha-diversity: Chao1 (pairwise Wilcoxon signed-rank *P* = 0.948, Friedman *P* = 1.000), Shannon (pairwise Wilcoxon signed-rank *P* = 0.267 Friedman, *P* = 0.607), and Simpson (pairwise Wilcoxon signed-rank *P* = 0.157, Friedman *P* = 1.000).

Panel B. Beta-diversity: Unweighted UniFrac matrix (PERMANOVA *P* = 0.997, comparison between samples, with up to 3 per participant). Principal coordinates (PC) 1 and 2 are the two axes that represent the most variability, shown in percentages. Ellipsoids show the 95% confidence intervals for the corresponding sample number (not available for the third sample procured due to the small number of participants involved).


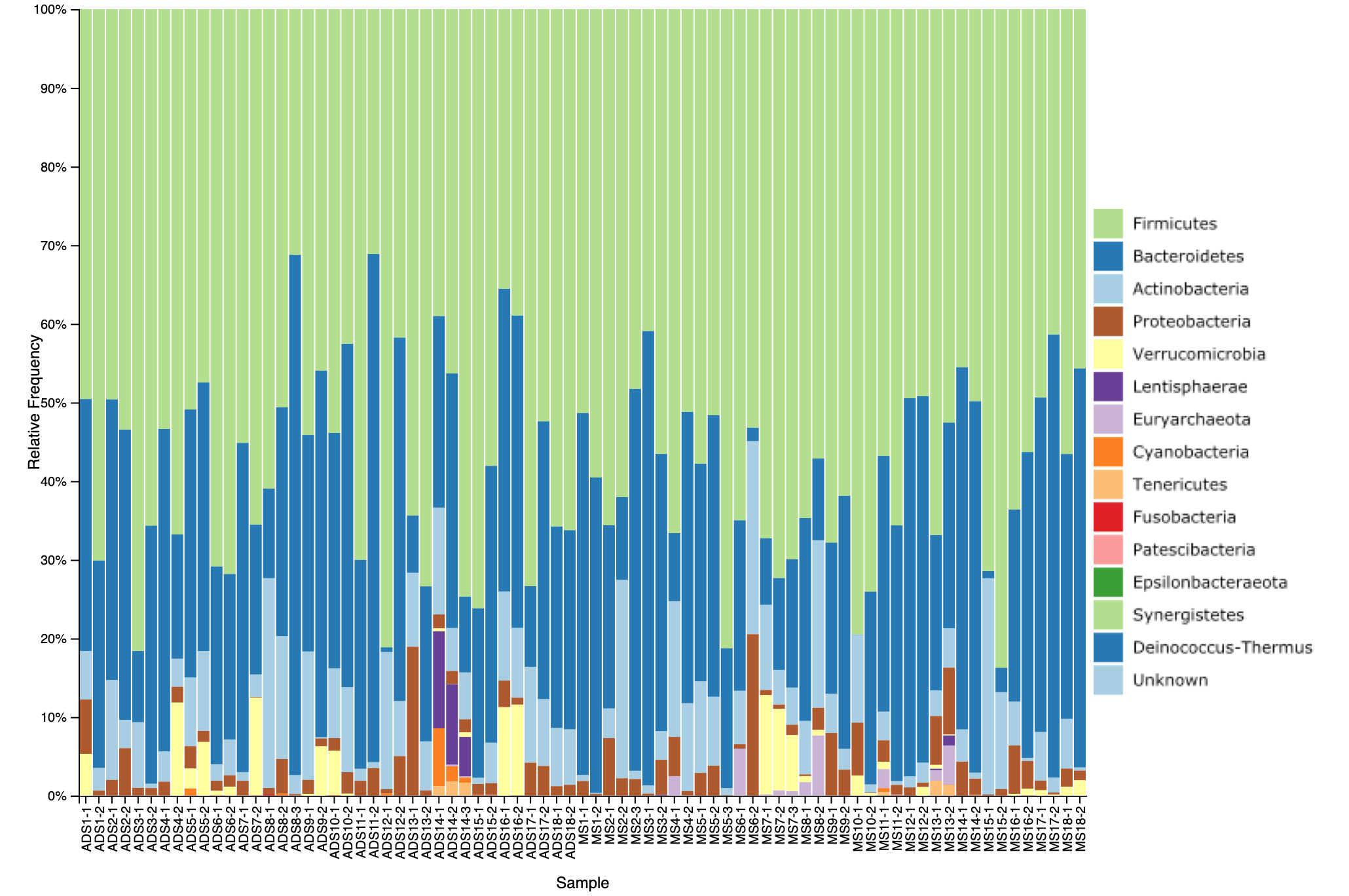


**Figure E3**. Phylum-level relative abundance depicted for individual stool samples procured from multiple sclerosis (MS) and monophasic acquired demyelinating syndrome (ADS) participants.

**Key**: n=36 participants contributed at least two stool samples, and five contributed three samples. Each individual participant with MS or ADS were assigned a number from 1 through to 18, which corresponds to the first number shown. The second suffix ‘-1’ denotes the first sample, ‘-2’ denotes the second sample, and ‘-3’ denotes the third sample. For example, MS 2-1, 2-2, and 2-3 represents the first, second and third sample from one individual with MS.


**Figure E4**. Genus-level non-parametric microbial interdependence test (NMIT) for comparison of longitudinal sample similarity between the multiple sclerosis (MS) and monophasic acquired demyelinating syndrome (mono ADS) cohorts (*P* = 0.289). Each coloured dot corresponds to the longitudinal average of up to 3 stool samples for each participant.

**Key**:

All participants (MS, n=18 and monoADS, n=18) contributed at least two stool samples, and five contributed three samples. For the entire cohort, the average time between samples one and two was 11.0 months, and between two and three was 8.2 months.

Axes 1 and 2 are the two axes that represent the most variability and are shown in percentages.


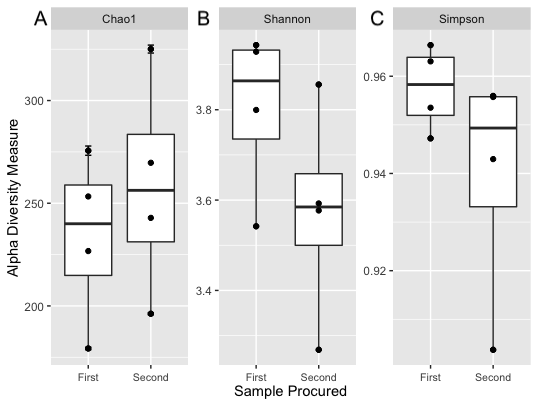


**Figure E5**. Alpha diversity of the gut microbiome over time for the multiple sclerosis participants who switched, stopped, or started a disease modifying therapy (DMT) between stool samples.

Panel A. Chao1 (pairwise Wilcoxon signed-rank *P* = 0.273). Panel B. Shannon (pairwise Wilcoxon signed-rank *P* = 0.144) and Panel (C. Simpson (pairwise Wilcoxon signed-rank *P* = 0.144). It remains possible that the observed differences in diversity between samples (a modest increase for Chao1 and decreases for Shannon and Simpson), while not statistically significant, could reach significance in a larger cohort.

For Panels A-C, n=4 of the MS participants had a change in their DMT status. All changes occurred between the first and second samples (mean time between these samples was 11.0 months). During this time, 1 switched from glatiramer acetate to rituximab, 1 stopped natalizumab, 1 started interferon beta-1a, and 1 started glatiramer acetate.

References

1. Mexhitaj I, Nyirenda MH, Li R, et al. Abnormal effector and regulatory T cell subsets in paediatric-onset multiple sclerosis. *Brain*. 2019;142(3):617-632.

2. Banwell B, Bar-Or A, Arnold DL, et al. Clinical, environmental, and genetic determinants of multiple sclerosis in children with acute demyelination: A prospective national cohort study. *Lancet neurol*. 2011;10(5):436-445.

3. Fadda G, Brown RA, Longoni G, et al. MRI and laboratory features and the performance of international criteria in the diagnosis of multiple sclerosis in children and adolescents: A prospective cohort study. *Lancet Child Adolesc Health*. 2018;2(3):191-204.

4. Hunsberger M, O'Malley J, Block T, Norris JC. Relative validation of block kids food screener for dietary assessment in children and adolescents. *Matern Child Nutr*. 2015;11(2):260-270.

5. Lewis SJ, Heaton KW. Stool form scale as a useful guide to intestinal transit time. *Scand J Gastroenterol*. 1997;32(9):920-924.

6. Pakpoor J, Seminatore B, Graves JS, et al. Dietary factors and pediatric multiple sclerosis: A case-control study. *Mult Scler*. 2018;24(8):1067-1076.

stylefix
